# Effects of BMI and Anticoagulant/Antiplatelet Therapy on the Severity of Gastrointestinal Bleeding

**DOI:** 10.3390/medicina62071390

**Published:** 2026-07-18

**Authors:** Melikşah Yüksel, Altuğ Şenol, Muhammet Cem Koçkar, Özgür Sirkeci, Naci Çil

**Affiliations:** 1 Department of Internal Medicine, Eskisehir City Hospital, 26080 Eskisehir, Turkey; 2 Department of Internal Medicine, Division of Gastroenterology, Faculty of Medicine, Suleyman Demirel University, 32260 Isparta, Turkey; senolaltug@gmail.com (A.Ş.); cemkockar@yahoo.com (M.C.K.); 3 Department of Internal Medicine, Division of Gastroenterology, Isparta City Hospital, 32200 Isparta, Turkey; ozgursirkeci@hotmail.com; 4 Department of Internal Medicine, Division of Gastroenterology, Burdur State Hospital, 15030 Burdur, Turkey; nacicil07@gmail.com

**Keywords:** obesity, BMI, anticoagulant, antiplatelet, gastrointestinal bleeding

## Abstract

*Background and Objectives:* Obesity is a major global public health problem associated with numerous complications. Likewise, the use of antiplatelet and anticoagulant agents has increased with population aging and the growing burden of cardiovascular diseases. This study aimed to evaluate the effects of obesity and antiplatelet/anticoagulant therapy on the clinical presentation, bleeding severity, and short-term clinical outcomes of patients with gastrointestinal bleeding. *Materials and Methods:* A prospective cohort study was conducted in consecutive patients admitted with upper or lower gastrointestinal bleeding to a tertiary referral center between January 2024 and January 2025. Patients were categorized according to body mass index (BMI) and antithrombotic therapy status. Clinical characteristics, bleeding severity scores, treatment requirements, and short-term clinical outcomes were compared. Multivariable multinomial logistic regression analysis was performed to identify independent associations. *Results:* A total of 177 patients were included. Obese and overweight patients were significantly older than normal-weight patients (*p* = 0.001), whereas no significant differences were observed among BMI groups regarding the Glasgow–Blatchford score, pre-endoscopic Rockall score, transfusion requirements, endoscopic intervention, or short-term clinical outcomes. Patients receiving antiplatelet or anticoagulant therapy had significantly higher Charlson Comorbidity Index, bleeding severity scores, and packed red blood cell transfusion requirements than those receiving no antithrombotic therapy (all *p* < 0.01). However, after multivariable adjustment, these associations did not remain independent, and advanced age remained the only independent factor common to both treatment groups. *Conclusions:* Obesity was not independently associated with bleeding severity or short-term clinical outcomes in patients with gastrointestinal bleeding. Although antiplatelet and anticoagulant therapy were associated with more severe bleeding-related clinical findings, these associations were not independent after multivariable adjustment.

## 1. Introduction

Gastrointestinal (GI) bleeding constitutes a critical medical emergency with significant morbidity and mortality, arising from hemorrhagic lesions located throughout the gastrointestinal tract. Clinically, GI bleeding is conventionally categorized based on the anatomic origin relative to the ligament of Treitz: bleeding arising proximal to this landmark is classified as upper GI bleeding, whereas hemorrhages emanating distal to it are designated as lower GI bleeding. This anatomical distinction is clinically pertinent, as it not only influences the spectrum of presenting symptoms but also determines the optimal diagnostic and therapeutic approach.

The advent and widespread use of anticoagulant and antiplatelet therapies have introduced additional complexity into the management of GI bleeding, markedly altering its epidemiology, clinical course, and associated risk profile. Empirical evidence underscores that patients receiving oral anticoagulants are at a substantially elevated risk of GI hemorrhage. For instance, Johnsen et al. conducted a large cohort study involving 4204 patients on oral anticoagulants, demonstrating a statistically significant increase in GI bleeding incidence compared to a control population without such pharmacotherapy [1]. Likewise, the hemorrhagic risk linked to aspirin monotherapy ranges from 0.7% to 1.3%, but this risk escalates when aspirin is combined with clopidogrel, with incidences reported between 1.2% and 2.0% [2]. In addition, Tsai et al. noted a higher prevalence of peptic ulcer disease among patients treated with clopidogrel relative to those prescribed aspirin, further implicating antiplatelet regimens in gastrointestinal mucosal vulnerability [3].

Parallel to pharmacological considerations, the global obesity epidemic represents a burgeoning public health challenge with profound implications for gastrointestinal pathology. Obesity is implicated as a significant risk factor for a spectrum of GI disorders, including erosive gastritis, esophageal and gastric malignancies, and acute pancreatitis [4,5,6]. Moreover, obesity is a well-recognized contributor to cardiovascular disease [7], thereby increasing the likelihood that affected individuals will necessitate long-term antithrombotic therapy. This intersection potentially amplifies both the incidence and severity of GI bleeding in this population.

Given these overlapping and interrelated risk factors, this study was designed to elucidate the influence of variations in body mass index (BMI) and concomitant use of antithrombotic agents on the clinical manifestation and outcomes of patients presenting with GI bleeding. Specifically, we sought to analyze differences in presenting symptoms, hemodynamic parameters, endoscopic findings delineating the nature of bleeding lesions, and the requirement for blood transfusion. Insight into these relationships is essential to optimize risk stratification and therapeutic strategies in this complex patient subset. Moreover, most studies investigating the association between obesity and GI bleeding have focused on patients with lower GI bleeding. In contrast, our study prospectively evaluated patients with both upper and lower GI bleeding within the same cohort.

## 2. Material and Methods

This prospective cohort study was conducted at the Gastroenterology Clinic of Süleyman Demirel University Faculty of Medicine, encompassing patient admissions between January 2024 and January 2025. The study population comprised individuals presenting with a presumptive diagnosis of gastrointestinal (GI) bleeding. Prior to inclusion, informed consent was obtained from all participants in accordance with institutional ethical standards and the Declaration of Helsinki.

Eligibility criteria included adults aged 19 years or older exhibiting clinical manifestations indicative of upper and/or lower GI bleeding. Patients were excluded if they were under 19 years of age, if they had undergone any gastrointestinal surgery within the preceding six weeks, or if the GI bleeding event manifested subsequent to hospital admission. Of the initial cohort of 236 patients, 177 met these criteria and were incorporated into the final analytical dataset.

Demographic and clinical data were collected for each participant, which included gender, chronological age, body mass index (BMI), and presenting symptoms categorized as melena, hematemesis, or hematochezia. Additionally, the presence of chronic comorbidities and active pharmacological therapies, specifically anticoagulant or antiplatelet agents, were documented. Upon admission, vital signs including heart rate and systolic and diastolic blood pressures (measured in mmHg) were obtained. Baseline laboratory investigations encompassed hemoglobin levels (g/dL), platelet count (×10^3^/mm^3^), coagulation profiles—prothrombin time (PT, seconds), international normalized ratio (INR), activated partial thromboplastin time (aPTT, seconds)—and serum urea concentration (mg/dL). Hematological, biochemical, and coagulation analyses were performed using the Beckman Coulter DxH 800 (Beckman Coulter, Brea, CA, USA), Siemens Atellica CH (Siemens Healthineers AG, Erlangen, Germany), and ACL TOP 700 (Instrumentation Laboratory, Bedford, MA, USA), respectively. To quantify patient comorbidity burden, the Charlson Comorbidity Index was calculated based on individual medical histories.

Key clinical outcomes were also followed, such as admission to the intensive care unit (ICU) and length of ICU stay. Detailed endoscopic evaluations were performed using a Fujinon EP-6000 endoscopy system (FUJIFILM Corporation, Tokyo, Japan), with findings categorized according to identified pathologies. Therapeutic interventions, both endoscopic and medical, were recorded alongside indications. Transfusion requirements were noted, specifying the types of blood products administered, including packed red blood cells, platelet apheresis, and fresh frozen plasma.

For the purpose of comparative analyses, patients were stratified into three BMI categories according to the National Institutes of Health (NIH) classification: normal weight (18.5–24.9 kg/m^2^), overweight (25.0–29.9 kg/m^2^), and obese (≥30 kg/m^2^). Patients with BMI below 18.5 kg/m^2^ were excluded from subgroup analyses due to insufficient sample size. Furthermore, participants were classified into three therapeutic cohorts based on their current antithrombotic regimens: (1) an anticoagulant group receiving agents such as heparin, enoxaparin, warfarin, rivaroxaban, edoxaban, apixaban, or dabigatran; (2) an antiplatelet group comprising individuals on monotherapy with either aspirin (brand name Ecopirin) or clopidogrel; and (3) a control group not receiving any antithrombotic therapy.

Comparative analyses were then performed across the BMI and treatment cohorts in order to evaluate variables, including gender distribution, presenting symptoms, Charlson Comorbidity Index scores, admission vital signs, baseline laboratory parameters, ICU admission rates, endoscopic findings, therapeutic modalities employed, and transfusion requirements.

### Statistical Analysis

Comparative analyses across independent groups were performed using appropriate statistical tests according to data distribution and measurement scale. Continuous variables meeting parametric assumptions were compared using One-Way Analysis of Variance (ANOVA), followed by Tukey’s Honestly Significant Difference (HSD) test for post hoc comparisons. Non-parametric continuous variables were analyzed using the Kruskal–Wallis test, followed by the Mann–Whitney U test when appropriate. Categorical variables were compared using Pearson’s chi-square test or Fisher’s exact test. Effect sizes were expressed as eta-squared (η^2^) for continuous variables and Cramér’s V for categorical variables. An a priori sample size calculation was performed using G*Power version 3.1.9.7 (Heinrich Heine University, Düsseldorf, Germany). For a one-way analysis of variance comparing three independent groups, assuming a medium effect size (Cohen’s f = 0.25), a significance level of 0.05, and a statistical power of 80%, the minimum required sample size was calculated as 159 patients.

Multinomial logistic regression analysis was performed to identify independent factors associated with BMI categories and antiplatelet/anticoagulant therapy groups. Variables that were statistically significant in the univariable analyses (*p* < 0.05) were entered into the multivariable models. Multicollinearity was assessed using tolerance and variance inflation factor (VIF) values, and variables showing evidence of multicollinearity or sparse data in categorical subgroups were excluded from the final models.

All statistical analyses were performed using IBM SPSS Statistics for Windows, version 22.0 (IBM Corp., Armonk, NY, USA). A two-sided *p*-value < 0.05 was considered statistically significant.

## 3. Results

### 3.1. Patient Characteristics

The study cohort comprised 177 patients with gastrointestinal (GI) bleeding, with a mean age of 62.6 ± 17.2 years. Of these, 102 (57.6%) were male and 75 (42.4%) were female. The mean body mass index (BMI) was 27.3 ± 3.0 kg/m^2^. Based on standardized BMI classifications, 21.5% of patients (*n* = 38) were categorized as normal weight, 63.3% (*n* = 112) as overweight, and 15.2% (*n* = 27) as obese.

Melena was the most common presenting symptom, occurring in 67.2% of patients (*n* = 119), followed by hematemesis (25.4%, *n* = 45) and hematochezia (16.9%, *n* = 30). Some patients presented with more than one symptom.

Regarding antithrombotic therapy, 61.0% of patients (*n* = 108) were not receiving antithrombotic treatment, 25.4% (*n* = 45) received antiplatelet therapy (aspirin or clopidogrel), and 13.6% (*n* = 24) received anticoagulant therapy (rivaroxaban, enoxaparin, warfarin, apixaban, dabigatran, or edoxaban).

### 3.2. Endoscopic Findings and Interventions

Endoscopic evaluation revealed a diverse spectrum of GI pathologies. The most frequent lesions identified included duodenal ulcers (17.5%, *n* = 31), gastric ulcers (14.1%, *n* = 25), and erosive gastritis (13.0%, *n* = 23). Additional findings comprised gastrointestinal arteriovenous malformations (11.3%, *n* = 20), GI cancers (6.8%, *n* = 12), gastritis and esophageal varices (each 6.2%, *n* = 11), esophageal ulcers (5.1%, n = 9), and esophagitis (4.5%, *n* = 8). Less common diagnoses included inflammatory bowel disease (3.4%, *n* = 6), concurrent gastric and duodenal ulcers (2.3%, *n* = 4), hemorrhoids (2.3%, *n* = 4), ileal ulcers, solitary rectal ulcers, and combinations of esophagitis with ulcers (each 1.1%, *n* = 2). Singular instances of bulbitis, duodenitis, and fundal varices were also documented.

Endoscopic therapeutic interventions were administered in 29.9% of patients (*n* = 53). Among these, argon plasma coagulation (APC) was the most commonly utilized modality (39.6%, *n* = 21), followed by sclerotherapy (34.0%, *n* = 18), band ligation (17.0%, *n* = 9), and a combination of sclerotherapy with hemoclip application (9.4%, *n* = 5). Medical management was provided to the majority of patients (97.2%, *n* = 172). Proton-pump inhibitor (PPI) monotherapy was employed in 87.2% (*n* = 150) of cases, while 15 patients (8.7%) received a combination of a PPI with somatostatin, and 7 (4.1%) received a PPI with vasopressors.

### 3.3. Comparison of Clinical Characteristics According to BMI Groups

Significant differences were observed in age, hematochezia, and NSAID use across BMI groups. Patients in the obese (68.6 ± 16.6 years) and overweight (64.0 ± 15.4 years) groups were significantly older than those in the normal-weight group (54.3 ± 19.9 years; *p* = 0.001). Hematochezia was more frequent in obese (25.9%) and overweight (18.8%) patients than in normal-weight patients (5.3%; *p* = 0.043). In contrast, NSAID use was more common among normal-weight (21.1%) and overweight (16.1%) patients, whereas no obese patient reported NSAID use (*p* = 0.023) (Table 1).

Heart rate and systolic blood pressure differed significantly across BMI groups. Normal-weight patients had a higher mean heart rate than overweight patients (*p* = 0.013), whereas systolic blood pressure was significantly higher in the overweight and obese groups than in the normal-weight group (*p* = 0.019). No significant differences were observed in the remaining vital signs or laboratory parameters (all *p* > 0.05) (Table 2).

### 3.4. Multinomial Logistic Regression Analysis According to BMI Groups

Multinomial logistic regression analysis was performed to identify factors independently associated with BMI categories. Variables that were significant in the univariable analyses (age, heart rate, systolic blood pressure, hematochezia, and NSAID use) were entered into the multinomial logistic regression model. Multicollinearity diagnostics showed no evidence of multicollinearity among the independent variables (all tolerance values > 0.10 and VIF values < 10). The model showed a good fit to the data (Pearson *p* = 0.854; Deviance *p* = 0.987) and explained 22.2% of the variance in BMI categories (Nagelkerke R^2^ = 0.222).

Age was the only variable independently associated with BMI categories. Each one-year increase in age was associated with a 2.9% higher likelihood of being overweight rather than normal weight (OR = 1.029, 95% CI: 1.01–1.05; *p* = 0.013) and a 4.6% higher likelihood of being obese rather than normal weight (OR = 1.046, 95% CI: 1.01–1.08; *p* = 0.009). Heart rate, systolic blood pressure, hematochezia, and NSAID use were not independently associated with BMI categories after multivariable adjustment (Table 3).

### 3.5. Comparison of Clinical Characteristics According to Antiplatelet and Anticoagulant Therapy Groups

Significant differences were observed in age, hematochezia, Charlson Comorbidity Index, Glasgow–Blatchford score, pre-endoscopic Rockall score, endoscopic treatment modality, packed red blood cell transfusion, and fresh frozen plasma transfusion across treatment groups. Patients receiving antiplatelet and anticoagulant therapy were significantly older than those receiving no antithrombotic therapy (*p* < 0.01). Hematochezia was more frequent in the antiplatelet (26.7%) and anticoagulant (25.0%) groups than in patients receiving no antithrombotic therapy (11.1%; *p* = 0.035). Post hoc analyses (Tukey’s HSD) demonstrated that patients receiving antiplatelet or anticoagulant therapy had a significantly higher Charlson Comorbidity Index, Glasgow–Blatchford score, and pre-endoscopic Rockall score than patients receiving no antithrombotic therapy (all *p* < 0.01). Endoscopic treatment modalities also differed significantly among treatment groups (*p* = 0.022). Packed red blood cell transfusion was more common in the antiplatelet (66.7%) and anticoagulant (66.7%) groups than in the no-therapy group (36.1%; *p* < 0.01), whereas fresh frozen plasma transfusion was most frequent in the anticoagulant group (20.8%; *p* = 0.005) (Table 4).

Significant differences were observed in diastolic blood pressure, hemoglobin, PT, INR, aPTT, and urea levels across treatment groups. Patients receiving anticoagulant therapy had significantly lower diastolic blood pressure than those receiving no antithrombotic therapy (*p* = 0.002). Hemoglobin levels were significantly lower in the antiplatelet group than in the no-therapy group (*p* = 0.005). PT, INR, and aPTT values were significantly higher in the anticoagulant group than in the other treatment groups (all *p* < 0.01). Urea levels were significantly higher in the antiplatelet and anticoagulant groups than in patients receiving no antithrombotic therapy (*p* < 0.01). No significant differences were observed in the remaining vital signs and laboratory parameters (all *p* > 0.05) (Table 5).

### 3.6. Multinomial Logistic Regression Analysis According to Antiplatelet and Anticoagulant Therapy Groups

Multinomial logistic regression analysis was performed to identify factors independently associated with antiplatelet and anticoagulant therapy groups. Variables that were statistically significant in the univariable analyses were entered into the model. Multicollinearity diagnostics showed no evidence of multicollinearity among the variables included in the final model. The model showed a good fit to the data (Pearson *p* = 0.664; Deviance *p *= 1.000) and explained 46.2% of the variance in treatment groups (Nagelkerke R^2^ = 0.462).

Age remained an independent predictor of both antiplatelet and anticoagulant therapy. Each one-year increase in age was associated with a 6.6% higher likelihood of receiving antiplatelet therapy rather than no antithrombotic therapy (OR = 1.066, 95% CI: 1.03–1.10; *p* < 0.001) and a 10.8% higher likelihood of receiving anticoagulant therapy (OR = 1.108, 95% CI: 1.05–1.17; *p* < 0.001). In addition, hematochezia was independently associated with antiplatelet therapy (OR = 3.789, 95% CI: 1.30–11.03; *p* = 0.015), whereas higher aPTT values were independently associated with anticoagulant therapy (OR = 1.078, 95% CI: 1.02–1.14; *p* = 0.006). No significant independent associations were observed for the remaining variables (Table 6).

## 4. Discussion

Our study demonstrated that obesity influenced the clinical presentation of gastrointestinal bleeding but had no significant impact on parameters reflecting bleeding severity or clinical management. Obese and overweight patients were older than normal-weight individuals and presented with higher systolic blood pressure and lower heart rate. However, no significant differences were observed among BMI groups regarding the Glasgow–Blatchford score, pre-endoscopic Rockall score, intensive care unit admission, requirement for endoscopic intervention, medical management, or blood transfusion. Multivariable regression analysis further demonstrated that age was the only factor independently associated with BMI categories.

Previous studies have consistently demonstrated that increasing BMI is associated with higher blood pressure and heart rate. Drøyvold et al. reported that higher BMI is an independent risk factor for hypertension, while Yadav et al. showed a positive correlation between BMI and heart rate [8,9]. Consistent with these findings, obese patients in our study had higher systolic blood pressure than normal-weight individuals. In contrast, mean heart rate was lower in obese patients, which may reflect the acute hemodynamic effects of gastrointestinal bleeding and early resuscitation, potentially masking the baseline cardiovascular effects of obesity.

Desai et al. investigated the impact of obesity in patients with lower gastrointestinal bleeding and found no significant difference in transfusion requirements between BMI groups; however, obese patients experienced more adverse clinical outcomes [10]. In contrast, our study demonstrated no significant differences in bleeding severity, as assessed by the Glasgow–Blatchford and pre-endoscopic Rockall scores, or in transfusion requirements across BMI groups. This discrepancy may be explained by differences in study design, as Desai et al. analyzed a large retrospective cohort limited to patients with lower gastrointestinal bleeding, whereas our study prospectively included patients with both upper and lower gastrointestinal bleeding.

Abougergi et al. reported that, among patients with non-variceal upper gastrointestinal bleeding, transfusion requirements did not differ significantly between obese and non-obese patients, whereas upper gastrointestinal endoscopy and endoscopic therapeutic interventions were performed more frequently in obese patients [11]. Similarly, we observed no significant difference in transfusion requirements across BMI groups. However, unlike the findings of Abougergi et al., the rate of endoscopic intervention was comparable among BMI groups in our cohort. This discrepancy may be attributable to differences in therapeutic strategies and the spectrum of endoscopic lesions identified in the two studies.

In a multicenter study of patients with acute lower gastrointestinal bleeding, Tominaga et al. reported that a high BMI was not associated with poor prognosis, whereas a low BMI (≤18.5 kg/m^2^) was identified as an independent predictor of both 30-day and 1-year mortality [12]. Similarly, in our study, increasing BMI was not associated with bleeding severity, Glasgow–Blatchford score, pre-endoscopic Rockall score, transfusion requirements, need for endoscopic intervention, or short-term clinical outcomes. These findings suggest that adverse clinical outcomes in patients with gastrointestinal bleeding may be more closely related to low BMI than to obesity. Nevertheless, because the number of patients with low BMI (≤18.5 kg/m^2^) was limited in our cohort, the impact of low BMI on clinical outcomes could not be adequately evaluated.

Another objective of the present study was to investigate the effects of antiplatelet and anticoagulant therapy on the clinical characteristics and bleeding severity of patients with gastrointestinal bleeding. Compared with patients who were not receiving antithrombotic therapy, those receiving antiplatelet or anticoagulant therapy were significantly older and presented more frequently with hematochezia. They also had a higher Charlson Comorbidity Index, Glasgow–Blatchford score, and pre-endoscopic Rockall score. Patients receiving anticoagulant therapy had lower diastolic blood pressure, whereas those receiving antiplatelet therapy had lower hemoglobin levels. In addition, packed red blood cell transfusion was more frequently required in both treatment groups, while fresh frozen plasma transfusion was significantly more common only among patients receiving anticoagulant therapy. No significant differences were observed among the groups regarding intensive care unit admission or medical management. In the multivariable regression analysis, the Charlson Comorbidity Index, Glasgow–Blatchford score, pre-endoscopic Rockall score, hemoglobin level, diastolic blood pressure, and packed red blood cell transfusion, which were significant in the univariable analyses, did not remain independently associated with treatment groups. Advanced age was the only independent factor common to both the antiplatelet and anticoagulant groups. Furthermore, hematochezia was independently associated only with antiplatelet therapy, whereas prolonged activated partial thromboplastin time (aPTT) was independently associated only with anticoagulant therapy.

One of the well-recognized consequences of the widespread use of antithrombotic agents is the increasing incidence of gastrointestinal bleeding [13,14]. Moreover, with increasing life expectancy and population aging, the use of antiplatelet and anticoagulant agents for the treatment and prevention of cardiovascular diseases continues to rise [15].

Hashash et al. reported that patients with lower gastrointestinal bleeding receiving antiplatelet or anticoagulant therapy were older, had a greater comorbidity burden, required packed red blood cell transfusions more frequently, experienced longer hospital stays, and had more severe bleeding than patients who were not receiving antithrombotic therapy [16]. In contrast, Božić et al. concluded that although antiplatelet and anticoagulant therapy increases the risk of gastrointestinal bleeding, it does not directly influence clinical outcomes once bleeding has occurred, and that prognosis is determined predominantly by patient-related factors [17]. More recently, Önem et al. evaluated elderly patients with lower gastrointestinal bleeding according to antiplatelet therapy, anticoagulant therapy, and no antithrombotic therapy, and found no significant differences in the need for endoscopic intervention, transfusion requirements, length of hospital stay, or 30-day mortality among these groups [18]. The authors emphasized that although antithrombotic therapy increases the risk of gastrointestinal bleeding, it does not independently determine the clinical course after bleeding has developed. Similarly, in our study, patients receiving antiplatelet or anticoagulant therapy had higher Glasgow–Blatchford and pre-endoscopic Rockall scores and required packed red blood cell transfusions more frequently in the univariable analyses. However, these associations did not remain significant after multivariable adjustment, with advanced age emerging as the only independent factor common to both treatment groups. These findings suggest that the unfavorable clinical characteristics observed in patients receiving antithrombotic therapy are more likely attributable to advanced age and accompanying patient-related factors than to the direct effects of the antithrombotic treatment itself.

Nevertheless, Hao et al., in a multicenter prospective study, identified both antiplatelet and anticoagulant therapy as independent risk factors for 28-day mortality [19]. In our cohort, although several indicators of bleeding severity were more pronounced among patients receiving antiplatelet or anticoagulant therapy in the univariable analyses, these associations did not remain independent after multivariable adjustment. This discrepancy may be explained by differences in study endpoints, as Hao et al. evaluated 28-day mortality as the primary outcome, whereas our study focused primarily on bleeding severity and short-term clinical characteristics.

Finally, hematochezia was independently associated with antiplatelet therapy in our study. However, no significant differences were observed in the distribution of endoscopically identified bleeding lesions that would suggest a higher prevalence of lower gastrointestinal bleeding among treatment groups. This finding may be explained by the inhibition of platelet aggregation caused by antiplatelet therapy, which impairs primary hemostasis and may render bleeding more clinically apparent as hematochezia. Nevertheless, further studies are required to clarify the underlying mechanisms of this association.

### Limitations and Future Prospects

This study has several limitations. First, although the study was prospectively designed, it was conducted at a single tertiary care center, and the relatively small sample sizes of the obese and anticoagulant therapy groups may have reduced the statistical power of some subgroup analyses. Second, despite performing multivariable regression analyses, residual confounding cannot be completely excluded because several potential confounding factors, including pre-admission proton-pump inhibitor use, *Helicobacter pylori* infection, alcohol consumption, previous gastrointestinal bleeding history, the severity of liver disease, and the detailed classification of antiplatelet and anticoagulant agents, were not evaluated. Third, patients with upper and lower gastrointestinal bleeding were analyzed together, which may have introduced heterogeneity into the study population. Finally, long-term clinical outcomes, such as rebleeding after hospital discharge and long-term mortality, were not assessed. Therefore, larger multicenter prospective studies with longer follow-up are warranted to validate our findings.

## 5. Conclusions

Obesity had no independent impact on bleeding severity, treatment requirements, or short-term clinical outcomes. Patients receiving antiplatelet or anticoagulant therapy exhibited clinical and prognostic indicators associated with greater bleeding severity; however, most of these associations were not confirmed to be independent of patient-related characteristics. These findings suggest that short-term clinical outcomes in patients with gastrointestinal bleeding are influenced more by patient-related factors, particularly advanced age, than by obesity, antiplatelet therapy, or anticoagulant therapy alone.

## Figures and Tables

**Table 1:** Comparison of Select Demographic and Clinical Variables Across BMI Groups.

Variable	Normal Weight (*N* = 38)	Overweight (*N* = 112)	Obese (*N* = 27)	Test Statistic (Effect Size, *p*-Value)
Gender, *n* (%)				χ^2^ = 2.988 V = 0.13 *p* = 0.224 ^a^
Female	19 (50.0)	42 (37.5)	14 (51.9)	
Male	19 (50.0)	70 (62.5)	13 (48.1)	
Age (years), *M* (*SD*)	54.3 (19.9)	64.0 (15.4)	68.6 (16.6)	F = 6.923 η^2^ = 0.074 *p* = 0.001 ^b^*
Melena, *n* (%)	27 (71.1)	73 (65.2)	19 (70.4)	*χ*^2^ = 0.587 V = 0.058 *p* = 0.746 ^a^
Hematemesis, *n* (%)	14 (36.8)	26 (23.2)	5 (18.5)	χ^2^ = 3.58 V = 0.142 *p* = 0.167 ^a^
Hematochezia, *n* (%)	2 (5.3)	21 (18.8)	7 (25.9)	χ^2^ = 5.89 V = 0.176 *p* = 0.043 ^c^
ICU Stay (days), Median (IQR)	2.5 (2.0–9.0)	1.0 (1.0–2.0)	2.0 (1.5–2.0)	χ^2^ = 5.946 η^2^ = 0.104 *p* = 0.051 ^d^
Received Endoscopic Therapy, *n* (%)	14 (36.8)	33 (29.5)	6 (22.2)	χ^2^ = 1.642 V = 0.096 *p* = 0.440 ^a^
Type of Endoscopic Therapy, *n* (%)				χ^2^ = 3.065 V = 0.161 *p* = 0.848 ^c^
Band ligation	3 (21.4)	6 (18.2)	0 (0.0)	
APC	4 (28.6)	14 (42.4)	3 (50.0)	
Sclerotherapy	6 (42.9)	10 (30.3)	2 (33.3)	
Sclerotherapy + hemoclip	1 (7.1)	3 (9.1)	1 (16.7)	
Received Medical Therapy, *n* (%)	38 (100.0)	109 (97.3)	25 (92.6)	χ^2^ = 2.717 V = 0.134 *p* = 0.172 ^c^
Type of Medical Therapy, *n* (%)				χ^2^ = 2.939 V = 0.102 *p* = 0.535 ^c^
PPI	31 (81.6)	95 (87.2)	24 (96.0)	
PPI + vasopressor	3 (7.9)	4 (3.7)	0 (0.0)	
PPI + somatostatin	4 (10.5)	10 (9.2)	1 (4.0)	
Transfusion: Packed Red Blood Cells, *n* (%)	21 (55.3)	50 (44.6)	14 (51.9)	χ^2^ = 1.469 V = 0.091 *p* = 0.480 ^a^
Transfusion: Fresh Frozen Plasma, *n* (%)	4 (10.5)	6 (5.4)	1 (3.7)	χ^2^ = 1.568 V = 0.096 *p* = 0.485 ^c^
Transfusion: Platelet Suspension, *n* (%)	1 (2.6)	3 (2.7)	0 (0.0)	χ^2^ = 0.510 V = 0.065 *p* = 1.000 ^c^
NSAID Use, *n* (%)	8 (21.1)	18 (16.1)	0 (0.0)	χ^2^ = 7.177 V = 0.185 *p* = 0.023 ^c^
Charlson Comorbidity Index, *M* (*SD*)	3.71 (3.30)	4.60 (3.41)	4.74 (3.07)	F = 1.140 η^2^ = 0.013 *p* = 0.322 ^b^
Glasgow–Blatchford Score, *M* (*SD*)	11.11 (4.03)	9.91 (3.43)	10.67 (3.41)	F = 1.783 η^2^ = 0.020 *p* = 0.171 ^b^
Pre-endoscopic Rockall Score, Median (IQR)	2 (1.0–5.0)	3 (1.0–4.0)	3 (1.0–5.0)	χ^2^ = 0.769 η^2^ = −0.007 *p* = 0.681 ^d^

*Note*. BMI = body mass index; *M* = mean; *SD* = standard deviation; V = Cramer’s V; η^2^ = Eta-squared; ICU = intensive care unit; IQR = interquartile range; APC = argon plasma coagulation; PPI = proton-pump inhibitor; NSAID = nonsteroidal anti-inflammatory drug. ^a^ Pearson Chi-square test. ^b^ One-Way ANOVA. ^c^ Fisher’s exact test. ^d^ Kruskal–Wallis test. * A significant result from the omnibus test prompted post hoc analysis with Tukey’s HSD.

**Table 2:** Comparison of Vital Signs and Laboratory Parameters Across BMI Groups.

Parameter	Normal Weight (*N* = 38)	Overweight (*N* = 112)	Obese (*N* = 27)	Test Statistic (η^2^, *p*-Value)
Heart Rate (beats/min), *M* (*SD*)	89.5 (18.1)	80.8 (15.0)	82.2 (13.9)	F = 4.442 η^2^ = 0.049 *p* = 0.013 ^a^*
Systolic Blood Pressure (mmHg), *M* (*SD*)	113.3 (18.5)	121.7 (17.6)	124.8 (19.5)	F = 4.050 η^2^ = 0.044 *p* = 0.019 ^a^*
Diastolic Blood Pressure (mmHg), *M* (*SD*)	67.4 (12.7)	70.3 (10.9)	73.0 (11.1)	F = 1.94 η^2^ = 0.022 *p* = 0.147 ^a^
Hemoglobin (g/dL), *M* (*SD*)	8.60 (2.96)	9.63 (2.56)	9.28 (2.22)	F = 2.221 η^2^ = 0.025 *p* = 0.112 ^a^
Platelet Count (×10^3^/mm^3^), Median (IQR)	224.5 (180.0–297.0)	224.0 (176.0–273.0)	240.0 (193.5–292.5)	χ^2^ = 0.952 η^2^ = −0.006 *p* = 0.621 ^b^
PT (s), Median (IQR)	12.7 (11.6–14.8)	12.7 (11.7–14.5)	13.0 (12.0–15.5)	χ^2^ = 0.20 η^2^ = −0.010 *p* = 0.906 ^b^
INR, Median (IQR)	1.10 (1.0–1.3)	1.10 (1.0–1.2)	1.10 (1.0–1.4)	χ^2^ = 0.133 η^2^ = −0.010 *p* = 0.936 ^b^
aPTT (s), Median (IQR)	27.0 (24.0–32.0)	29.0 (27.0–32.0)	28.0 (26.5–29.0)	χ^2^ = 4.174 η^2^ = 0.012 *p* = 0.124 ^b^
Urea (mg/dL), Median (IQR)	43.5 (30.0–74.0)	43.0 (31.0–62.0)	55.0 (37.5–75.0)	χ^2^ = 5.878 η^2^ = 0.022 *p* = 0.053 ^b^

*Note*. BMI = body mass index; *M* = mean; *SD* = standard deviation; IQR = interquartile range; η^2^ = Eta-squared; PT = prothrombin time; INR = international normalized ratio; aPTT = activated partial thromboplastin time. ^a^ One-Way ANOVA. ^b^ Kruskal–Wallis Test. * A significant result from the omnibus test prompted post hoc analysis with Tukey’s HSD.

**Table 3:** Multinomial Logistic Regression Analysis for BMI Categories.

BMI Category	Variable	*B*	*SE*	Wald *χ*^2^	*p*	OR (Exp(B))	95% CI
Overweight	Age	0.028	0.012	6.109	0.013	1.029	1.01–1.05
	Heart Rate	−0.026	0.014	3.578	0.059	0.975	0.95–1.01
	Systolic Blood Pressure	0.019	0.012	2.512	0.113	1.019	0.99–1.04
	Hematochezia	1.420	0.795	3.186	0.074	4.135	0.87–19.66
	NSAID Use †	0.232	0.537	0.188	0.665	1.262	0.44–3.61
Obese	Age	0.045	0.017	6.868	0.009	1.046	1.01–1.08
	Heart Rate	−0.013	0.018	0.544	0.461	0.987	0.95–1.02
	Systolic Blood Pressure	0.029	0.016	3.361	0.067	1.029	0.99–1.06
	Hematochezia	1.678	0.882	3.618	0.057	5.356	0.95–30.19
	NSAID Use †	−20.030	<0.001	–	–	–	–

*Note*. Reference category for the dependent variable: Normal weight. *B* = regression coefficient; *SE* = standard error; OR = odds ratio; CI = confidence interval; NSAID = nonsteroidal anti-inflammatory drug. † Perfect separation occurred because no obese patient reported NSAID use; therefore, the Wald statistic and *p* value could not be estimated.

**Table 4:** Comparison of Clinical Characteristics Across Antiplatelet and Anticoagulant Therapy Groups.

Variable	No Antithrombotic Therapy (*N* = 108)	Antiplatelet Therapy (*N* = 45)	Anticoagulant Therapy (*N* = 24)	Test Statistic (Effect Size, *p*-Value)
Gender, *n* (%)				χ^2^ = 0.315 V = 0.042 *p* = 0.854 ^a^
Female	44 (40.7)	20 (44.4)	11 (45.8)	
Male	64 (59.3)	25 (55.6)	13 (54.2)	
Age (years), *M* (*SD*)	56.5 (17.2)	70.7 (12.1)	74.9 (12.1)	F = 22.311 η^2^ = 0.204 *p* < 0.01 ^b^*
Melena, *n* (%)	75 (69.4)	29 (64.4)	15 (62.5)	χ^2^ = 0.643 V = 0.060 *p* = 0.725 ^a^
Hematemesis, *n* (%)	32 (29.6)	7 (15.6)	6 (25.0)	χ^2^ = 3.321 V = 0.137 *p* = 0.190 ^a^
Hematochezia, *n* (%)	12 (11.1)	12 (26.7)	6 (25.0)	χ^2^ = 6.881 V = 0.195 *p* = 0.035 ^c^
ICU Stay (days), Median (IQR)	2.0 (1.0–3.5)	1.0 (1.0–8.5)	2.0 (2.0–2.0)	χ^2^ = 0.223 η^2^ = −0.047 *p* = 0.894 ^d^
Received Endoscopic Therapy, *n* (%)	34 (31.5)	15 (33.3)	4 (16.7)	χ^2^ = 2.385 V = 0.116 *p* = 0.303 ^a^
Type of Endoscopic Therapy, *n* (%)				χ^2^ = 12.360 V = 0.338 *p* = 0.022 ^c^
Band ligation	8 (23.5)	0 (0.0)	1 (25.0)	
APC	9 (26.5)	10 (66.7)	2 (50.0)	
Sclerotherapy	13 (38.2)	5 (33.3)	0 (0.0)	
Sclerotherapy + hemoclip	4 (11.8)	0 (0.0)	1 (25.0)	
Received Medical Therapy, *n* (%)	107 (99.1)	43 (95.6)	22 (91.7)	χ^2^ = 4.696 V = 0.159 *p* = 0.058 ^c^
Type of Medical Therapy, *n* (%)				χ^2^ = 3.576 V = 0.098 *p* = 0.454 ^c^
PPI	93 (86.9)	39 (90.7)	18 (81.8)	
PPI + vasopressor	6 (5.6)	0 (0.0)	1 (4.5)	
PPI + somatostatin	8 (7.5)	4 (9.3)	3 (13.6)	
Transfusion: Packed Red Blood Cells, *n* (%)	39 (36.1)	30 (66.7)	16 (66.7)	χ^2^ = 15.748 V = 0.298 *p* < 0.01 ^a^
Transfusion: Fresh Frozen Plasma, *n* (%)	6 (5.6)	0 (0.0)	5 (20.8)	χ^2^ = 9.696 V = 0.259 *p* = 0.005 ^c^
Transfusion: Platelet Suspension, *n* (%)	2 (1.9)	2 (4.4)	0 (0.0)	χ^2^ = 1.325 V = 0.095 *p* = 0.610 ^c^
NSAID Use, *n* (%)	19 (17.6)	5 (11.1)	2 (8.3)	χ^2^ = 1.612 V = 0.105 *p* = 0.480 ^c^
Charlson Comorbidity Index, *M* (*SD*)	3.48 (2.97)	5.44 (3.36)	6.79 (3.31)	F = 14.277 η^2^ = 0.141 *p* < 0.01 ^b^
Glasgow–Blatchford Score, *M* (*SD*)	9.40 (3.51)	11.38 (3.14)	12.17 (3.46)	F = 9.519 η^2^ = 0.099 *p* < 0.01 ^b^
Pre-endoscopic Rockall Score, *M* (*SD*)	2.17 (2.01)	3.64 (1.46)	4.25 (1.26)	F = 19.406 η^2^ = 0.182 *p* < 0.01 ^d^

*Note*. *M* = mean; *SD* = standard deviation; ICU = intensive care unit; IQR = interquartile range; V = Cramer’s V; η^2^ = Eta-squared; APC = argon plasma coagulation; PPI = proton-pump inhibitor; NSAID = nonsteroidal anti-inflammatory drug. ^a^ Pearson Chi-square test. ^b^ One-Way ANOVA. ^c^ Fisher’s exact test. ^d^ Kruskal–Wallis test. * A significant result from the omnibus test prompted post hoc analysis with Tukey’s HSD.

**Table 5:** Comparison of Vital Signs and Laboratory Parameters Across Anticoagulant and Antiplatelet Therapy Groups.

Parameter	No Antithrombotic Therapy (*N* = 108)	Antiplatelet Therapy (*N* = 45)	Anticoagulant Therapy (*N* = 24)	Test Statistic (η^2^, *p*-Value)
Heart Rate (beats/min), *M* (*SD*)	83.0 (16.4)	82.7 (16.0)	82.4 (13.7)	F = 0.019 η^2^ = 0.000 *p* = 0.982 ^a^
Systolic Blood Pressure (mmHg), *M* (*SD*)	122.0 (16.8)	119.0 (17.7)	115.6 (25.0)	F = 1.351 η^2^ = 0.015 *p* = 0.262 ^a^
Diastolic Blood Pressure (mmHg), *M* (*SD*)	72.3 (11.0)	67.8 (9.4)	64.5 (14.2)	F = 6.263 η^2^ = 0.067 *p* = 0.002 ^a^*
Hemoglobin (g/dL), *M* (*SD*)	9.87 (2.67)	8.52 (2.13)	8.63 (2.74)	F = 5.559 η^2^ = 0.060 *p* = 0.005 ^a^*
Platelet Count (×10^3^/mm^3^), Median (IQR)	226.0 (180.0–271.5)	224.0 (176.0–276.0)	248.0 (198.0–316.5)	χ^2^ = 1.751 η^2^ = 0.001 *p* = 0.417 ^b^
PT (s), Median (IQR)	12.2 (11.6–13.8)	13.0 (11.6–14.5)	17.0 (14.3–30.0)	χ^2^ = 25.837 η^2^ = 0.137 *p* < 0.01 ^b^**
INR, Median (IQR)	1.00 (0.97–1.20)	1.10 (1.00–1.24)	1.45 (1.23–2.55)	χ^2^ = 28.524 η^2^ = 0.152 *p* < 0.01 ^b^**
aPTT (s), Median (IQR)	29.0 (26.0–31.5)	28.0 (25.0–30.0)	33.5 (29.0–39.0)	χ^2^ = 13.797 η^2^ = 0.068 *p* = 0.001 ^b^**
Urea (mg/dL), Median (IQR)	36.5 (29.0–55.5)	57.0 (43.0–71.0)	48.0 (38.5–88.0)	χ^2^ = 17.836 η^2^ = 0.091 *p* < 0.01 ^b^**

*Note*. *M* = mean; *SD* = standard deviation; η^2^ = Eta-squared; IQR = interquartile range; PT = prothrombin time; INR = international normalized ratio; aPTT = activated partial thromboplastin time. ^a^ One-Way ANOVA. ^b^ Kruskal–Wallis Test. * A significant result from the omnibus ANOVA prompted post hoc analysis with Tukey’s HSD. ** A significant result from the omnibus Kruskal–Wallis test prompted post hoc analysis with the Mann–Whitney U test.

**Table 6:** Multinomial Logistic Regression Analysis of Factors Associated with Antiplatelet and Anticoagulant Therapy Groups.

Therapy Group	Variable	*B*	*SE*	Wald *χ*^2^	*p*	OR (Exp(B))	95% CI
Antiplatelet therapy	Age	0.064	0.018	12.154	<0.001	1.066	1.03–1.10
	Charlson Comorbidity Index	−0.068	0.096	0.497	0.481	0.934	0.77–1.13
	Glasgow–Blatchford Score	0.060	0.112	0.288	0.591	1.062	0.85–1.32
	Pre-endoscopic Rockall Score	0.098	0.188	0.270	0.603	1.103	0.76–1.59
	Diastolic Blood Pressure	−0.029	0.022	1.824	0.177	0.971	0.93–1.01
	Hemoglobin	−0.016	0.151	0.011	0.915	0.984	0.73–1.32
	aPTT	−0.044	0.039	1.224	0.269	0.957	0.89–1.03
	Urea	−0.003	0.006	0.211	0.646	0.997	0.98–1.01
	Hematochezia	1.332	0.545	5.975	0.015	3.789	1.30–11.03
	Packed Red Blood Cell Transfusion	0.675	0.603	1.254	0.263	1.964	0.60–6.40
Anticoagulant therapy	Age	0.102	0.028	12.987	<0.001	1.108	1.05–1.17
	Charlson Comorbidity Index	0.081	0.127	0.409	0.522	1.085	0.84–1.39
	Glasgow–Blatchford Score	0.295	0.157	3.548	0.060	1.344	0.99–1.83
	Pre-endoscopic Rockall Score	−0.162	0.296	0.298	0.585	0.851	0.48–1.52
	Diastolic Blood Pressure	−0.044	0.029	2.319	0.128	0.957	0.91–1.01
	Hemoglobin	−0.067	0.198	0.114	0.736	0.935	0.64–1.38
	aPTT	0.075	0.027	7.542	0.006	1.078	1.02–1.14
	Urea	−0.010	0.009	1.333	0.248	0.990	0.97–1.01
	Hematochezia	1.381	0.752	3.373	0.066	3.978	0.91–17.36
	Packed Red Blood Cell Transfusion	0.026	0.781	0.001	0.973	1.026	0.22–4.74

*Note*. Reference category for the dependent variable: No antithrombotic therapy. *B* = regression coefficient; *SE* = standard error; OR = odds ratio; CI = confidence interval; aPTT = activated partial thromboplastin time.

## Data Availability

The data presented in this study are available on request from the corresponding author. The data are not publicly available due to privacy and ethical restrictions.
